# Thermally-Conductive and Mechanically-Robust Graphene Nanoplatelet Reinforced UO_2_ Composite Nuclear Fuels

**DOI:** 10.1038/s41598-018-21034-4

**Published:** 2018-02-14

**Authors:** Tiankai Yao, Guoqing Xin, Spencer Michael Scott, Bowen Gong, Jie Lian

**Affiliations:** Department of Mechanical, Aerospace and Nuclear Engineering, Rensselaer Polytechnic Institute, Troy, NY 12180 USA

## Abstract

Low thermal transport behavior along the radial direction of nuclear fuel pellets and pellet-cladding mechanical interaction significantly impact fuel performance and the safety of current nuclear energy systems. Here we report a new strategy of advanced fuel design in which highly thermally-conductive and mechanically-robust graphene nanoplatelets are incorporated into UO_2_ fuel matrix to improve fuel thermal-mechanical properties. The 2D geometry of the graphene nanoplatelets enables a unique lamellar structure upon fuel consolidation by spark plasma sintering. The thermal conductivity along the radial direction of the sintered fuel pellets at room temperature reaches 12.7 and 19.1 wm^−1^K^−1^ at 1 wt.% and 5 wt.% loadings of the graphene nanoplatelets, respectively, representing at least 74% and 162% enhancements as compared to pure UO_2_ fuel pellets. Indentation testing suggests great capability of the 2D graphene nanoplatelets to deflect and pin crack propagation, drastically improving the crack propagation resistance of fuel matrix. The estimated indentation fracture toughness reaches 3.5 MPa·m^1/2^ by 1 wt.% loading of graphene nano-platelets, representing a 150% improvement over 1.4 MPa·m^1/2^ for pure UO_2_ fuel pellets. Isothermal annealing of the composite fuel indicates that the graphene nano-platelet is able to retain its structure and properties against reaction with UO_2_ matrix up to 1150 °C.

## Introduction

Advanced fuels with enhanced safety margin and accident tolerance are of vital importance for the safe operation of current light water reactor fleets and the development of future advanced nuclear energy systems. The current UO_2_ fuel for LWRs has low thermal conductivity which will further be reduced upon burnup. The low thermal transfer efficiency results in a large temperature gradient within the fuel pellet, high centerline temperature, and thermal stress. The fuel cladding mechanical interaction and fission product retention capability limit the lifetime of UO_2_ fuel pins in the reactor, presenting the great challenges for extending fuel burn-up, effective utilization of nuclear resources, and minimization of nuclear waste accumulation. Breakthrough technologies of advanced fuel designs are necessary that can greatly improve thermal-mechanical properties and thus enhanced fuel performance under both normal operation and beyond design conditions.

Various concepts of accident tolerance fuels (ATFs) have been proposed such as a fully ceramic microencapsulated (FCM) fuel^1^, high fissile density fuels^2,3^, and oxide fuels with additives^4,5^. Highly thermally-conductive secondary phases, such as SiC and carbon nanotubes, were incorporated into UO_2_ fuel pellets to increase thermal conductivity. For instance, a UO_2_–10 vol.% silicon carbide (SiC) composite pellet shows a 62% improvement of thermal conductivity as compared with pure UO_2_^6^, and UO_2_-5 vol.% carbon nanotube shows 29.7% increase in thermal conductivity^5^. Graphene, a 2D single sheet of carbon atoms bonding in a hexagonal arrangement, has extremely high in-plane thermal conductivity (3000 W m^−1^ K^−1^)^7^ and mechanical stiffness (1060 GPa)^8^, and thus is a potential filler as the reinforced component of composite fuels to improve their thermal-mechanical properties. A theoretical study^9^ shows UO_2_-graphene composite fuels have a better performance that monolithic UO_2_ fuels due to the potential benefits of incorporating highly thermally-conductive graphene nano-platelet with low neutron absorption cross section.

In this paper, UO_2_ composite fuel pellets with various graphene nano-platelet (hereafter referred as GNP) loadings from 1 wt.% to 5 wt.% are consolidated into dense fuel pellets by spark plasma sintering (SPS) showing a unique lamellar interconnection along in-plane direction and thus improved thermal diffusivity/conductivity along the radial direction of the fuel matrix. Incorporation of mechanically-robust graphitic nanoplatelets significantly increases the resistance of the fuel pellets against cracking propagation upon mechanical loadings and indentation. The stability of GNP upon sintering and reacting with surrounding UO_2_ matrix is also explored and GNP can be well preserved up to 1150 °C in a helium atmosphere.

## Result and Discussion

**Figure 1:**
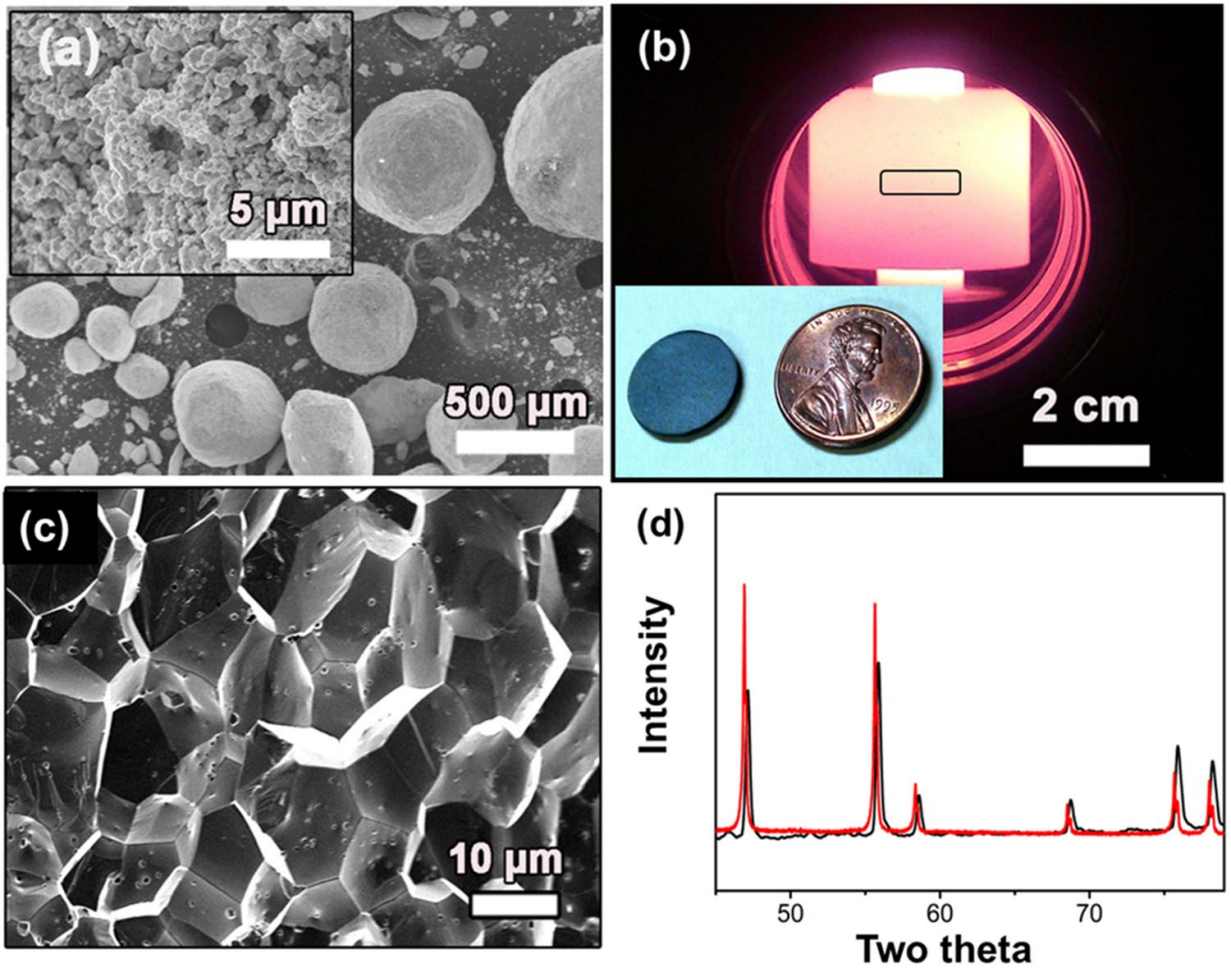
(**a**) Starting powders are several hundred µm agglomerates of submicron UO_2 + x_ crystallites; (**b**) a photo of SPS sintering apparatus with a black box showing where powder compact is located during sintering; Inserted in the lower left corner is the sintered pellet by SPS at 1500 °C for 5 mins with a US penny for comparison; (**c**) A cross section fractured surface showing the sintered UO_2_ pellet with an average grain size of 12 ± 1 µm in an equiaxial shape; (**d**) XRD patterns showing no phase change before (black lines) and after (red lines) SPS process. However, a shift of peaks from high 2θ to lower ones indicates the concurrent reduction and sintering of UO_2 + x_ powder by SPS.

Pure UO_2_ pellets with a theoretical density (TD) of 98.36% (10.79 g/cc) were prepared by SPS at 1500 °C for 5 mins under an applied pressure of 40 MPa in a vacuum atmosphere from as procured powder (Fig. 1a). Figure 1b shows a typical photocopy of the sintered pellet, and the thickness of the sintered pellet is around 0.5 mm. Cross-section SEM images of the fracture surface (Fig. 1c) shows the information of grain size, pore shape, and pore distribution. The average size of the equiaxial UO_2_ grains is 12 ± 1 µm, which falls into the typical grain size range of 10–15 µm for commercial LWRs UO_2_ fuels fabricated by conventional sintering^10^.

XRD patterns indicate no phase change before and after SPS (Fig. 1d); however, there is an appreciable peak shift from higher angles to lower ones. For example, the UO_2_ (111) diffraction peak (2θ) shifts from 28.45° of the starting powder to 28.22° of the sintered pellets. These results indicate the capability of SPS graphite dies to *in-situ* reduce the hyper-stoichiometric starting powders to a lower degree of oxygen enrichment during sintering. Calculation using Braggs’ law from peaks from 20 to 90° yields a lattice parameter of 5.473 ± 0.001 Å, closely in line with the value of 5.472 ± 0.009 Å reported in the literature for stoichiometric UO_2_^11,12^, and indicates the pellet sintered by SPS at 1500 °C for 5 mins is very close to stoichiometric UO_2_. Hyper-stoichiometric UO_2_ powder was claimed to be reduced by graphite dies during SPS process^11^, attributing to the reduction of U^5+^ and U^6+^ into U^4+^ with a larger ionic radius resulting in lattice expansion and XRD peak shifts to lower angles according to Braggs’ law.

**Figure 2:**
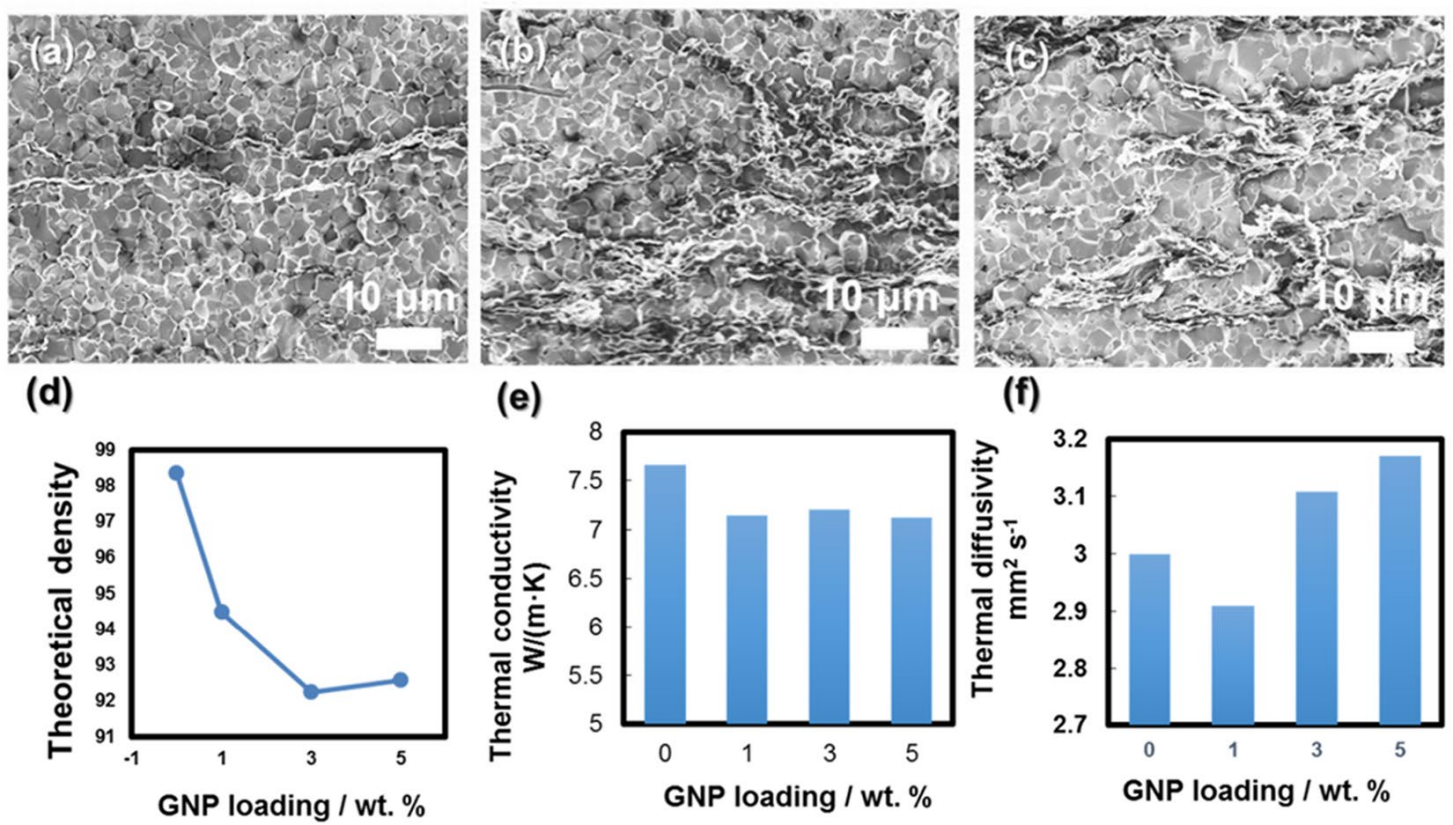
Microstructure of UO_2_-GNP composite fuel pellets with different graphene loadings: 1 wt.% (**a**), 3 wt.% (**b**), and 5 wt.% (**c**) sintered by SPS at 1600 °C for 8 mins; the dependence of theoretical density (**d**); cross-plane thermal diffusivity (**e**) and thermal conductivity (**f**) as a function of GNP loadings.

The incorporation of GNP suppresses the sintering kinetics in which GNP can act as physical barriers^13–15^ to pin atomic diffusion and grain boundary migration during sintering and thus reduces physical density and average grain size of the sintering pellets. Figure 2a–c show microstructure of the UO_2_ composite fuel pellets with different GNP loadings (1, 3, and 5 wt.%) upon consolidation by SPS at 1600 °C for 8 mins (herein referred as 1G-, 3G- and 5G-1600-8 pellets, respectively). For 1G-1600-8 pellets, the physical density (Fig. 2d) and grain size are reduced to ~94% TD and 3~5 µm, respectively, and the density is further reduced to 92–93% for 3 G and 5G-1600-8 pellets. A longer duration (e.g., 20 mins) or higher sintering temperatures slightly promote the densification (e.g., ~95% TD for 5G-1600-20) and significant grain size coarsening does not occur (see supplementary data). There is also a slight reduction of the cross-plane thermal diffusivity for 1G-1600-8 pellets which can be attributed to interface thermal resistance accross the GNP platelets/oxide matrix  and lack of the interconnection of the GNP along this direction. In general, no significant variation in cross-plane thermal diffusivity and conductivity (Fig. 2e,f) is observed for GNP-loaded UO_2_ composite fuel pellets within experimental uncertainties of the laser flash measuring technique.

**Figure 3:**
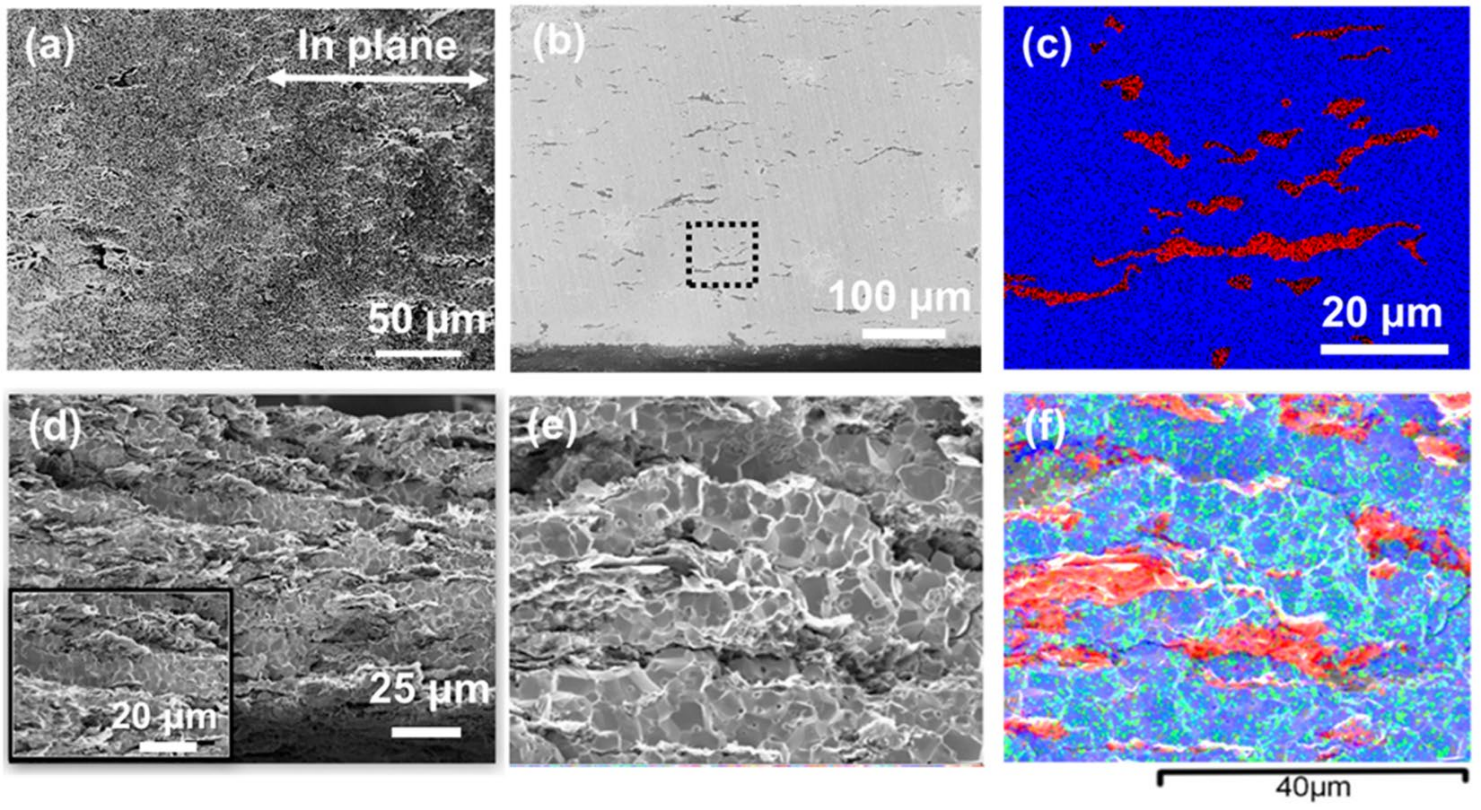
Microstructure of fractured (**a**) and polished (**b**) cross section surfaces of the UO_2_-1G pellets with EDS mapping overlay (**c**, blue for U and red for C element); A fractured cross section view of the UO_2_-5G pellets (**d**); with detailed microstructure (**e**) and corresponding element mapping (**f**).

The incorporation of the highly thermally-conductive and mechanically-strong graphene nanoplatelets greatly enhance the thermal-mechanical properties of the sintered composite fuel pellets, particularly on the thermal transport along the radial direction due to a unique laminar structure. Both fractured (Fig. 3a) and polished (Fig. 3b) cross section surfaces of the sintered composite fuel pellets (1G-1600-8) indicate a laminated microstructure with GNP layers continuously interconnected mainly along in-plane direction in the UO_2_-1G pellets. Similar features are also found in the UO_2_-5G pellets (5G-1600-20) but with a more evident laminated microstructure and more connection of GNP along radial direction (Fig. 3d,e). Those features are further verified by EDS mappings (Fig. 3c–f) with alternating layers of U and C elements. In UO_2_-5G pellets, element mapping (Fig. 3f) suggests the UO_2_ between GNPs is ~15 μm thick, and the grain size is ~3 μm. The effect of sintering temperature and holding time on density is provided in Fig. S1 (supplementary information) for the UO_2_-5G pellets.

**Figure 4:**
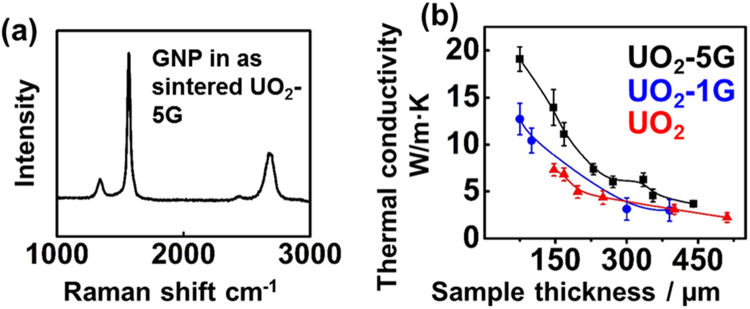
(**a**) A Raman spectrum of GNP in as-sintered UO_2_-5G pellets shows its high crystallinity after sintering; (**b**) in-plane thermal conductivity of the sintered UO_2_ and UO_2_-GNP composite fuel pellets. The in-plane thermal conductivity was performed with a setup shown in the Supplementary Fig. S2. The pellets were polished down to different sample thickness in order to accurately determinate the true in-plane thermal conductivity. The thinner sample thickness for testing, the closer of the in-plane thermal conductivity due to the minimized interference from cross plane thermal transport. As a result of highly thermally conductive GNP in the UO_2_-GNP composite fuel pellets, the measured in-plane thermal conductivity of UO_2_-GNP increase accordingly at greater GNP loading.

Raman spectrum (Fig. 4a) shows a dominated graphite band and a single intense 2D band at 2700 cm^−1^ and a low I _D_/I _G_ ratio (0.14), indicating that the structural integrity of GNP is well preserved in the composite fuel pellets upon SPS densification process^16^. The unique laminated structure and well-preserved GNP suggest the possibility of increasing the in-plane thermal conductivity of sintered composite fuel pellets. To minimize the influence of thermal transport in across plane direction on in-plane thermal diffusivity measurements, we conducted the measurement on polished thin slices with various thicknesses using an in-plane holder^17^ (see Fig. S2 in supplementary information). It is expected that the in-plane thermal conductivity increases monotonically with reduced sample thickness due to the minimization of the heat conduction along cross-plane direction. Therefore, thinner pellets for laser flash measurement more accurate measurement of the in-plane thermal conductivity. A reverse thickness-dependent trend was observed for in-plane thermal conductivity with composite fuel slices, which outperforms pure ones at all thicknesses (Fig. 4b). For example, when sample thickness is decreased to 150 µm, the thermal conductivity of the pure UO_2_ pellet (7.28 W/M·K) approaching very closely to the value measured at cross-plane direction (7.80 W/M·K, data not shown here) and literature reported values^18,19^. At this thickness, the thermal conductivity of the UO_2_-5G pellets is 13.94 W/M·K, ~90% higher than pure UO_2_.

Further thickness reduction by mechanical polishing leads to the fragmentation of the pure UO_2_ pellets due to its brittle nature. In contrast, the UO_2_-GNP composite fuel pellets display greatly-enhanced resistance against mechanical polishing-induced cracks, indicating the superior ductile character of composite fuel pellets. At the thickness of 75 µm, the sample length (5 mm) for heat transport along the in-plane direction is 66 times of the sample thickness, and an in-plane thermal conductivity approaching to the true value can be obtained. The in-plane thermal conductivities of UO_2_-1G and UO_2_-5G pellets along the radial direction are 12.72 and 19.09 W/m·K, respectively, representing over ~74% and 162% improvements of thermal conductivity, respectively, as compared to pure bulk UO_2_. Note that the true in-plane thermal conductivities for the composite fuel pellets could be even higher since the thermal diffusivity measurement may still be affected by the temperature gradient across-plane through the thickness (75 microns). Additionally, the layered structure (Fig. 3f) has a alternative arragnement of UO_2_ grain laminates (~15 µm) and GNP layers (~3 µm). For more accurate measurement of the true in-plane thermal diffusivity of the composite fuel pellets, it is better to polish the samples down to ~20 µm thick containing only one layer of GNP at the center, which is difficult to achieve a proper geometry for laser flash measurement with a uniform thickness.

**Figure 5:**
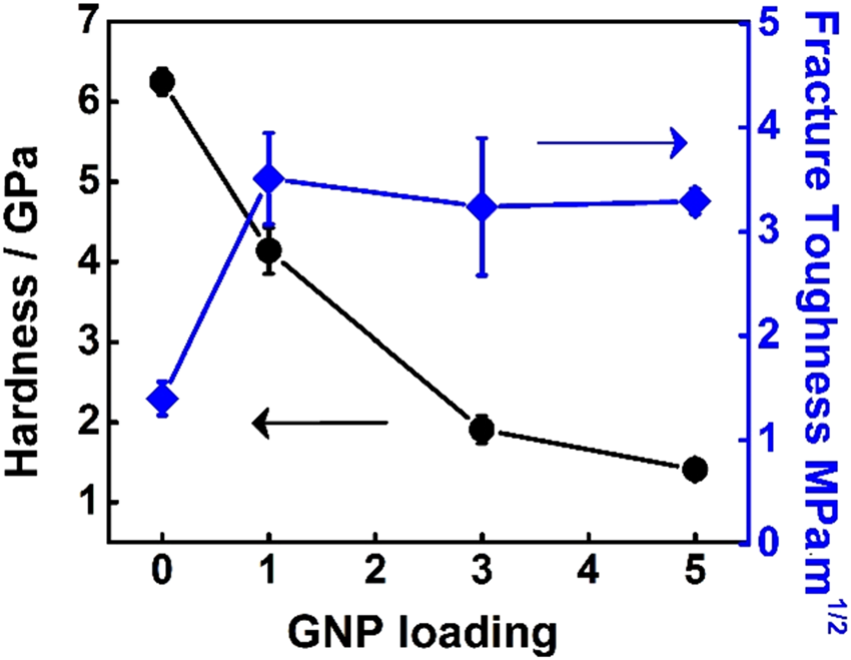
Micro-hardness and estimated fracture toughness of the sintered pellets as a function of GNP loading.

**Figure 6:**
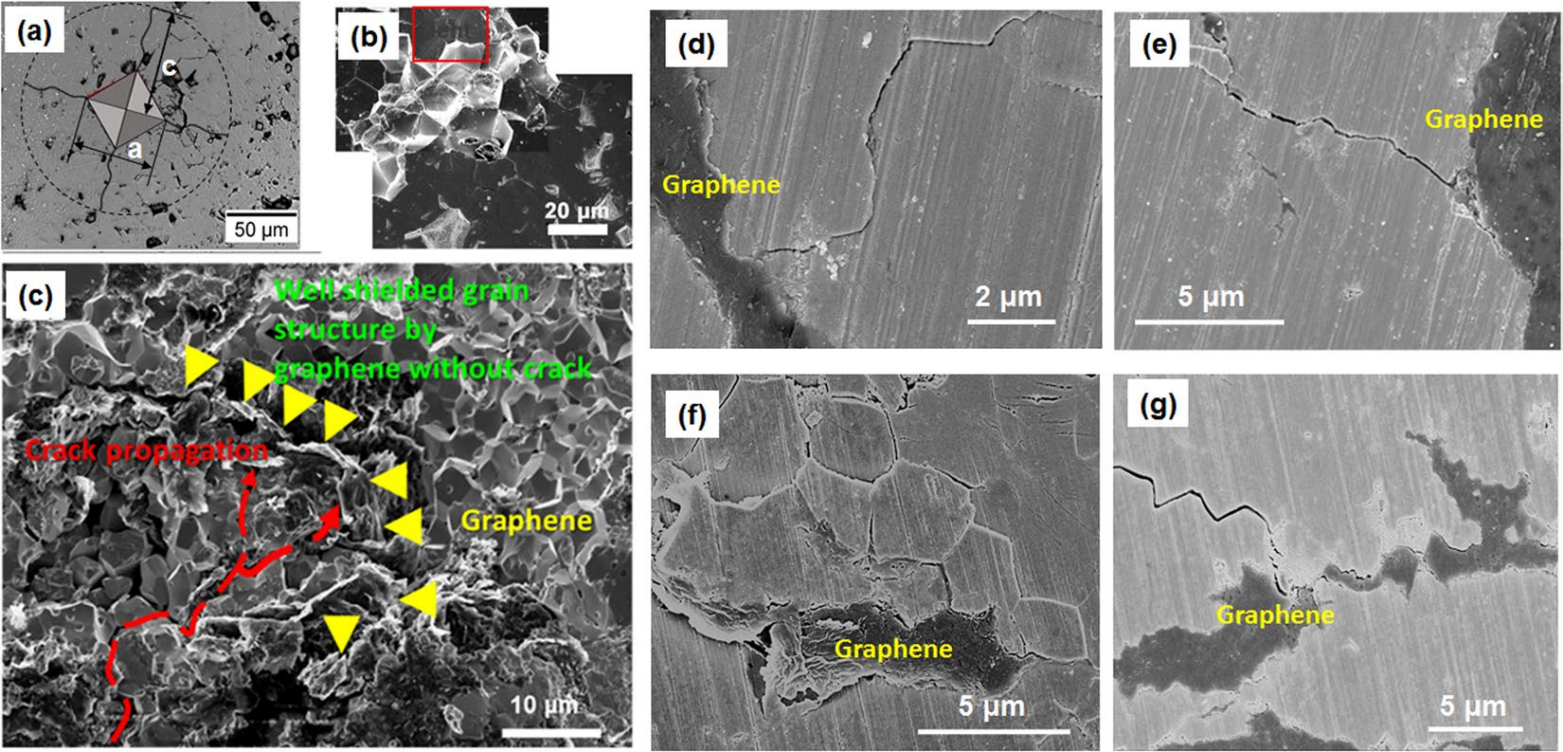
(**a**) A scheme imposed on an optical microscopy image of an indent on the pure UO_2_ pellet with marked *a* and *c*, two dimensional parameters used for fracture toughness estimation; (**b**)SEM images show the indentation induced cracks propagating along grain boundaries in the pure UO_2_ pellet, resulting in grain spallation and clearly visible grain boundaries; Red box shows where the indent sits; (**c**) On contrary, the cracks in the UO_2_-GNP composite fuel pellets can be effectively harnessed by GNP, leaving grain structure behind GPN intact; (**d**–**f**) SEM images further show crack tips effectively pined and deflected by GNP.

The development of advanced fuels with enhanced thermal-mechanical properties capable of effective heat transfer and minimizing fuel pellet mechanical interaction (FCMI) is crucial for improving the reactor operation safety margin and accident tolerance. Oxide fuels experience FCMI due to volumetric swelling of the fuel pellets upon burning and incorporation of the fission products. The FCMI exerts enormous stress on the cladding tubes, leading to the high temperature creep and perforation of cladding and eventually failure of fuel rods. With increasing graphene loadings, the hardness of the composite fuel pellets decreases drastically (Fig. 5), implying that the composite fuels may display greatly enhanced plasticity and fracture toughness, beneficial for minimizing the FCMI. The estimated fracture toughness, based on indentation technique (Fig. 6), of pure UO_2_ pellets is 1.4 ± 0.2 MPa·m^1/2^, which matches very well with previously reported values^20–23^ (Table 1). With the incorporation of 1 wt.% GNP, there is an appreciable increase of fracture toughness to 3.5 ± 0.4 MPa·m^1/2^, an enhancement of 1.5 times than pure UO_2_. Further increase of GNP loading does not lead to continuous improvement.

**Table 1:** Thermal-mechanical properties of proposed accident tolerant and standard UO_2_ fuel pellets.

Sample condition	Thermal conductivity (W/m·K)	Hardness (GPa)	Fracture toughness (MPa·M^1/2^)
UO_2_^this work^	7.28 ± 0.02	6.3 ± 0.2	1.4 ± 0.2
UO_2_-1 wt.% GNP ^this work^	12.7 ± 1.7	4.1 ± 0.3	3.5 ± 0.4
UO_2_-5 wt.% GNP ^this work^	19.1 ± 1.4	1.4 ± 0.1	3.30 ± 0.1
0.5 wt.% TiO_2_ doped UO_2_^28^	7.8	6.4 ± 0.6	—
UO_2_-5 vol. % diamond^29^	9.6	—	—
UO_2_-10 vol. % SiC^39^	10.5	—	—
0.05-1% Al(OH)_3_ doped UO_2_^30^	9.8	—	—
Pure UO_2_^40^	—	6.0 ± 0.4	—
Ti_2_O_3_ doped UO_2_^20^	—	5.26–6.70	0.88–1.14
UO_2_^21^	—	6.4 ± 0.5	1.1 ± 0.2
UO_2_^22^	—	—	0.88–1.20
UO_2_^23^	—	—	1.012 ± 0.025
UO_2_^18^, nuclear fuel pellet	6.5–8.5	—	—
UO_2_ single crystal <100> direction^19^	7.59	—	—
UO_2_ single crystal <110> direction^19^	8.53	—	—
UO_2_ single crystal <111> direction^19^	8.44	—	—

The GNP-UO_2_ composite fuel pellets also shows excellent resistance to crack generation and propagation as compared with the pure UO_2_ pellets. Indentation test was performed on the pure UO_2_ (Fig. 6a,b) and UO_2_-1G composite fuel pellets (Fig. 6c–g). For the pure UO_2_ fuel pellet, an indentation induced crack propagates freely along grain boundaries. Some grains located on the propagating path were spalled off, leaving the grain boundary structures clearly visible by SEM imaging (Fig. 6b). On the contrary,  the UO_2_-1G pellets show a better capability to harness cracks at the interface of UO_2_ grains and GNP as revealed by Fig. 6d–g, GNP perfectly shield underneath grains with a pristine grain structure well preserved without damage. Cracks can be effectively deflected and harnessed without further propagation when encountering graphene nanoplatelets. This shielding effect could be related to shear deformable GNP redistributing crack energy and preventing further crack propagation, demonstrating great potentials of the highly mechanically robust and flexible 2D graphene nanoplatelets as reinforced agents of the composite fuels to improve mechanical properties and crack propagation resistance.

**Figure 7:**
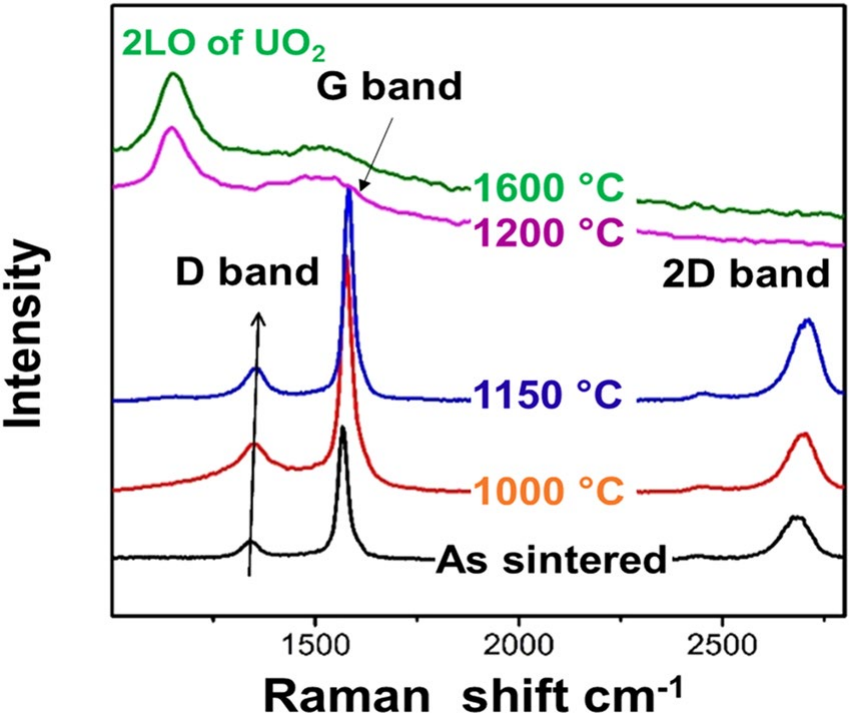
Raman spectra of the GNP in as-sintered UO_2_-5G pellet after isothermal annealing at various temperatures for 10 hours in helium atmosphere.

The structural integrity of GNP in the composite fuel pellets was also investigated by post-sintering thermal treatment. As an allotropy of carbonaceous materials, graphene may react with oxide fuels to form UC and release CO, degrading their performance of the composite fuel pellets as a potential fuel candidate. Previous studies indicated that pre-compacted high-density UO_2_-carbon black mixture pellet begins to release CO gas at ~1200 K^24^. As such, extended isothermal treatment (10 hours) at various temperature ranges from 1000 °C to 1600 °C were conducted in a helium atmosphere to thermally simulate in-pile working condition. After thermal annealing, Raman spectrum (Fig. 7) shows unchanged D/G ratios up to 1150 °C in a helium atmosphere, indicating the GNP in the composite fuel pellets remains intact. Above this temperature, characteristic peaks for GNP disappears with merging of the T_2g_ band for UO_2_. However, the proposed formation of uranium carbide is not visible at Raman spectrum since it is Raman inactive^25^. A shift of Raman active bands for GNP with isothermal holding temperatures up to 1200 °C, indicating an interaction between the uranium and GNP possibly through either electron doping or strain effect^26^. Recent study^27^ shows prolonged SPS holding at hours during sintering can *in-situ* thermally induce graphitisation of graphene within Al_2_O_3_-graphene nanocomposite and improve oxidation resistance and thermal stability over the short SPS consolidation (mins). This finding implies structural stability of UO_2_-graphene composite fuel pellets can be further improved by prolonging the sintering duration and improving graphene crystallinity.

Table 1 summarized the comparison of the thermal-mechanical properties of the advanced fuel design^6,28–30^. Among them, the UO_2_-GNP composite fuel form prepared in this study shows the greatest promise with a simultaneously improved thermal conductivity and fracture toughness at a minimum loading of GNP (Note, the 1 wt.% GNP is 4.6 vol.% if density of graphite, 2.26 g/cm^3^ is used for GNP). A recent study^9^ of neutronics and fuel performance modeling using MARS-KS reported that the UO_2_/graphene composite fuel is capable of lowering the fuel centerline temperature from 2100 k to 1700 K, due to a 33.26% enhancement of thermal conductivity from 10 vol.% graphene. Given the significant improvement (74% to 162%) on thermal conductivity, a significant reduction of center line temperature is expected, enabling stable 2D graphene nanoplatelets in the composite fuel. The greatly enhanced thermal-mechanical properties underscore the innovative concept of fuel design using highly thermally-conductive and mechanically robust 2D graphene as reinforced agents of composite fuels to improve fuel performance and accident tolerance. On the other hand, vigorous in-pile testing under relevant reactor conditions is needed to validate fuel performance of the GNP-UO_2_ composite fuels and quantify the new fuel forms for potential applications.

## Conclusion

UO_2_-GNP composites were developed by SPS, in which highly thermally-conductive and mechanically-strong graphene nanoplatelets were used as fillers to improve the thermal-mechanical properties and accident tolerance of nuclear fuels. A unique lamellar structure was obtained with the 2D graphene nanosheets aligned along the radial direction of the sintered fuel pellets. A 162% improvement of thermal conductivity in the radial direction (in-plane) is achieved for the UO_2_-5 wt.% GNP composite fuel sintered at 1600 °C for 20 mins under a uniaxial pressure of 40 MPa. Furthermore, composite fuel pellets show greatly-enhanced capability to resist crack propagation. GNP within the as sintered UO_2_-5 wt.% graphene composite fuel pellet is thermally stable up to 1150 °C in helium atmosphere for 10 hrs. Due to the greatly enhanced thermal transport along the radial direction and the capability against crack propagation, the UO_2_-GNP composite fuel may show great potentials as a candidate to reduce the center line temperature, enhance safety margin of the fuel system, minimize FCMI and thus significantly improve fuel performance.

## Methods

### Powder and Pellet fabrication

Graphene nanoplatelets are fabricated from thermal exfoliation and reduction of graphite oxide powders and high-temperature annealing at 2200 °C^31^. Pure UO_2_ powders as shown in Fig. 1a were procured from International Bio-analytical Industries Inc. (Boca Raton, Florida, USA) with a typical particle feature of hundreds of microns–sized agglomerates consisting of sub-micron sized UO_2_ crystallites. UO_2_-GNP powder mixtures were pre-processed by high energy ball milling (HEBM) (Fritsch, Pulverisette 7, Idar-Oberstein, Germany) thorugh a half hour dry milling at a speed of 500 rpm using 10 mm WC balls. Sintering of the composite fuel pellets (Fig. 1b) was conducted by SPS using a Dr. Sinter® SPS-211 Lx system. The HEBMed powders were loaded into graphite dies and sintered at 1500–1600 °C and various durations from 5 to 20 mins. The heating rate was 100 °C/min before 1200 °C and then reduced to 50 °C/min. A uniaxial hydraulic pressure of 40 MPa was applied on the powder compact through graphite dies at the beginning of sintering, and released right after cooling process started. The density of sintered pellets was measured based on Archimedes method using an Adam analytical scale system (Danbury, NY, USA) with distilled water as the immersing medium. A detailed process information can also be found in ref.^28^.

### Phase and microstructure characterization

Phase composition and crystallinity of the sintered pellets were investigated by X-ray diffraction using a Panalytical X’ Pert (Westborough, MA, USA) diffractometer (Cu_Ka_ = 1.5406 Å). Carl Xeiss Supra (Jena, Germany) field emission Scanning electron microscopy (SEM) was used to characterize microstructure features of the sintered pellets, mainly UO_2_ grain size and GNPs distribution.

### Thermal properties measurement

Thermal diffusivity of the sintered pellets was measured by a laser flash apparatus (LFA-457, NETZCH). For cross-plane measurement, a 8 mm square sample was cut from the sintered pellet along the in-plane direction by a low-speed diamond saw and grinded and polished down to ~0.5 mm thick. For in-plane measurement, samples were loaded into a special sample holder^32–34^ which allows the measurement of *t*_1/2_ takes for temperature to raise to peak value at a distance of ~5.4 mm away from the center of laser spot (for a detailed description of the holder and method, please refer to the Fig. S2 of supplementary materials). Both surfaces of the polished pellets are coated with graphite spray before the test to eliminate the influence of infrared signal reflection. The measurement was conducted in argon inert atmosphere at a flow rate of 150 ml/min. Half time for temperature raise was restricted to be shorter than 200 ms. Cape-Lehman + pulse correction was used to fit the signal – time curve to derive thermal diffusivity values. Thermal conductivity can be calculated as the product of density, thermal diffusivity, and specific thermal capacity. The densities of the sintered fuel pellets are measured on a scale system based on Archimedes principle at room temperature. The specific heat capacities of pristine UO_2_, UO_2_-1G, and UO_2_-5G pellets are calculated based on the rule of mixture. A value of 0.237 J/g·K is used for UO_2_^35^; while 0.711 J/g·K, the value for graphite, is used for GNP since the values for the two carbon materials are identical above 100 K^36^.

### Mechanical properties measurement

Mechanical properties, including hardness and fracture toughness, of sintered fuel pellet was investigated by Vickers microhardness indentation technique (LECO 400 FT) on polished sample surface with final step polishing using 0.5 μm diamond film. Hardness values were obtained by using small loading of 100 g for 10 s holding at the peak value. Fracture toughness was estimated by typical cracking method using the following equation^37^:

$${K}_{IC}=0.016(\frac{E}{H})0.5(\frac{P}{{c}^{1.5}})$$

where *K*_*IC*_ is estimated fracture toughness; *H* is measured hardness; E is the elastic modulus of UO_2_ with a value of 220 GPa^38^, *P* is the applied loading used to initiate the cracking which is 1 Kg; and *c* is measured crack length from the center of indent produced by a 15 s holding at the peak loading of 1 Kg.

### Thermal stability assessment

Post-sintering structural thermal stability of the composite fuel pellets was evaluated by annealing the sintered pellets at various temperatures (1000 °C, 1100 °C, 1150 °C, 1200 °C, 1400 °C and 1600 °C) in helium inert atmosphere for 10 hours. Raman spectrum (Alpha300R, WItec, Germany) with 532 nm laser source was utilized to study structural integrity of GNPs at room temperature after thermal annealing.

## Electronic supplementary material

Supplementary information
