# Supplementary material for: Thermally-Conductive and Mechanically-Robust Graphene Nanoplatelet Reinforced UO2 Composite Nuclear Fuels
Source: Sci Rep. 2018 Feb 14;8:2987. doi: 10.1038/s41598-018-21034-4 (PMC5812999; doi:10.1038/s41598-018-21034-4)
Supplement: Supplementary file 1 — Supplementary information [file 41598_2018_21034_MOESM1_ESM.pdf]

Supplementary information for "Thermal-mechanically Robust Graphene Nanoplatelet reinforced UO_2 Composite Nuclear Fuels"

Tiankai Yao, Guoqing Xin, Spencer M. Scott, Bowen Gong, Jie Lian*

Department of Mechanical, Aerospace, and Nuclear Engineering, Rensselaer Polytechnic Institute, Troy, NY 12180, USA

* *Corresponding author; Email: lianj@rpi.edu*

Supplementary Information

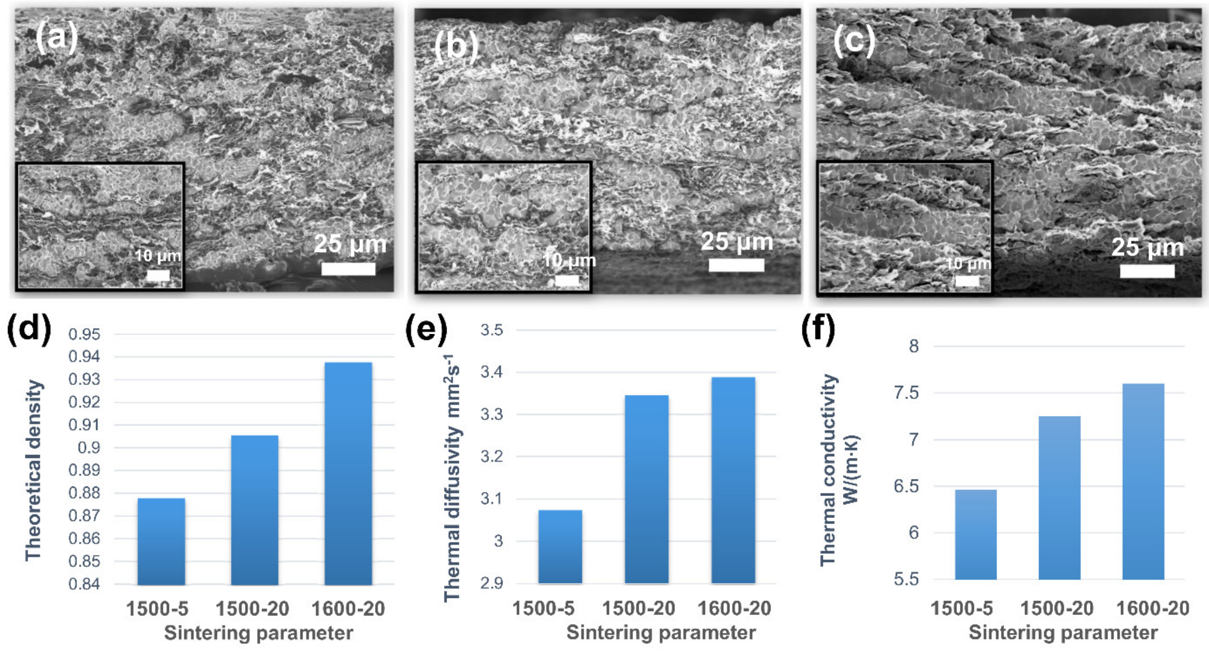

Fig. S1. Microstructure features for UO_2 pellet with 5 wt. % GNP loading sintered at 1500 °C for 5 mins (a); 1500 °C for 20 mins; and 1600 °C for 20 mins. Measured pellet density (d), thermal diffusivity (e), and calculated thermal conductivity increases slightly with elongated sintering holding time and higher sintering temperature

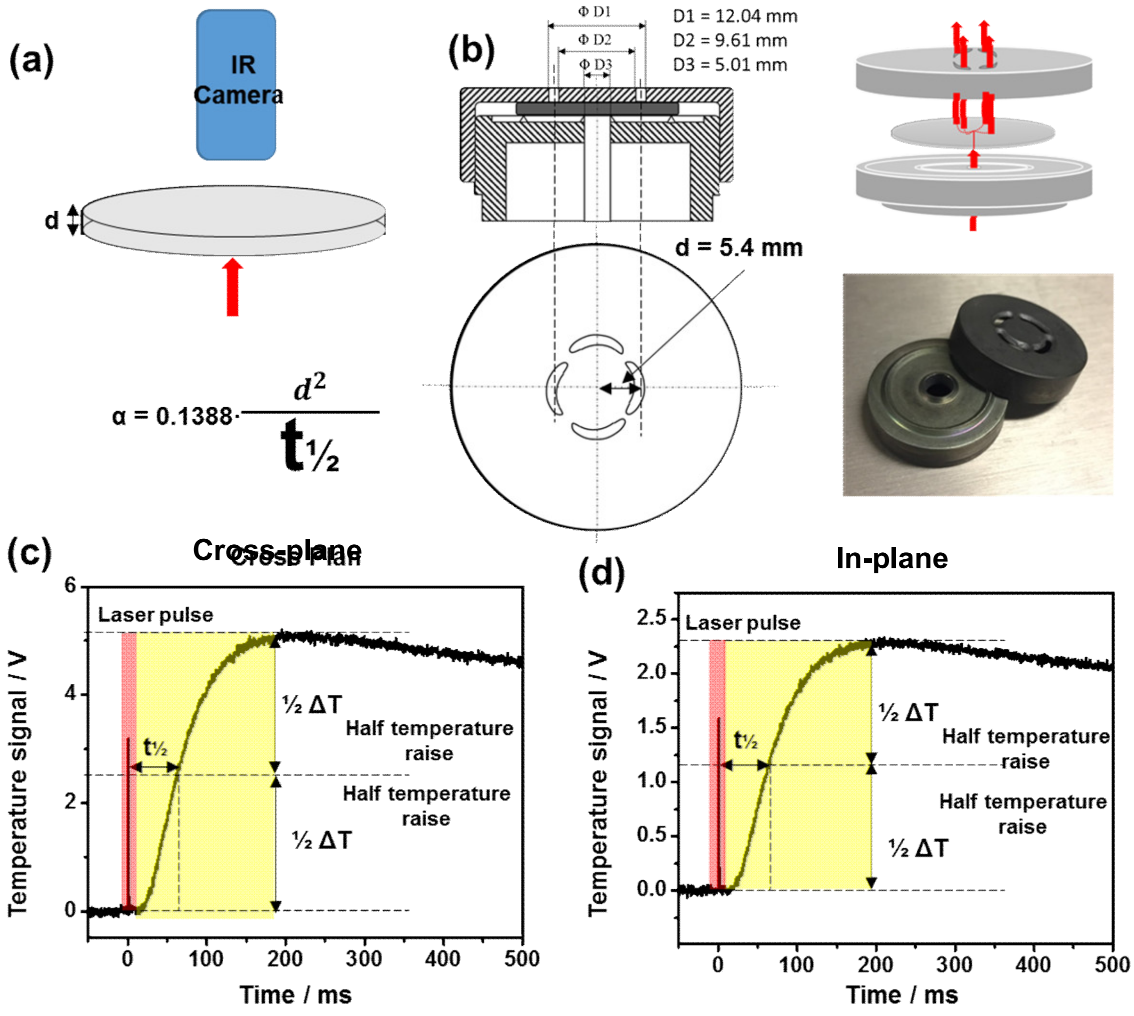

Fig. S2 (a) scheme of laser flash method principle in which IR camera is used to record how long it takes for half raise of the temperature of upside of sample after the downside was heated by a pulsed laser, the equation used to calculate thermal diffusivity is also presented in which d is the sample thickness. (b) shows the scheme and photocopy of the in-plane holder shows *d* is fixed as 5.4 mm. (c) and (d) shows the measured temperature raise profile vs time for 5G sample in cross plane model at a a thickness of 1.27 mm and in plane model at thickness of 75 µm. The similarity of two curves in terms of half time for temperature raise demonstrate the high fidelity of the method used here to for in-plane thermal diffusivity measurement.
